# Relationships between physical activity, body image, BMI, depression and anxiety in Chinese college students during the COVID-19 pandemic

**DOI:** 10.1186/s12889-022-14917-9

**Published:** 2023-01-05

**Authors:** Bing Han, Guoli Du, Yashu Yang, Jiping Chen, Guoxiao Sun

**Affiliations:** 1 School of Physical Education, Shandong University, Jinan, Shandong China; 2 School of Philosophy and Social Development, Shandong University, Jinan, Shandong China

**Keywords:** Depression, Anxiety, Body image, BMI, Physical activity, College students

## Abstract

**Background:**

Both depression and anxiety are worldwide burden that is not being abated with our current knowledge and treatment of the condition. Numerous clinical trials have supported that physical activity (PA) can reduce the depression and anxiety in adolescents, but little is known about its mechanism of action. Therefore, the study objectives were to explore the potential relationship between physical activity and depression and anxiety from the perspective of body image and body mass index (BMI), and to provide an important reference for future self-esteem education and health promotion intervention.

**Methods:**

The participants in this study were 251 Chinese college students between 17 and 22 years old. Participants completed the International Physical Activity Questionnaire-Short Form (IPAQ-SF), the Body Image Questionnaire (BIQ), the Self-rating Depression Scale (SDS) and the Self-rating Anxiety Scale (SAS). A descriptive and correlational approach was used, using the PROCESS macro for Statistical Package for the Social Sciences (SPSS).

**Results:**

(1) Physical activity was significantly negatively correlated with both depression and anxiety (t = -0.216, *p* < 0.001; t = -0.184, *p* < 0.01). (2) Body image had a significant moderating effect on the relationship between physical activity and anxiety among college students, but there was no moderating effect between depression and physical activity. BMI has no moderating effect on the two interrelationships.

**Conclusion:**

There is only body image that moderates the relationship between anxiety and physical activity.

## Background

Depression and anxiety are the most common psychiatric conditions, they often bring serious inner pain to their sufferers and bring serious burdens to their families [1, 2]. The WHO has predicted that depression would be the second largest contributor to the global burden of disease by 2020 [3], and it is clear that this prediction has come true. As a common concomitant of depression, anxiety is not far behind—374 million people in the world suffer from it [4]. The novel coronavirus epidemic is an international public health emergency. Since December 2019, the spread of the COVID-19 epidemic in China and elsewhere in the world has led citizens to be urged to stay at home or significantly restrict their outdoor activities, which could harm their psychological well-being [5, 6]. College students’ mental health has grown in popularity as a public concern in recent years. According to the Household Pulse Study released by the United States Census Bureau, ratios of anxiety and depression in college-age students have soared through the pandemic [7]. Current research shows that about 24.9% of Chinese college students reported having anxiety symptoms [8], while the overall prevalence rate of depression is 23.8% [9]. Reports on suicides among college students have also attracted social attention [10].

College is the key stage before college students enter society. It is an important period for their values and personality to form [11]. However, although the researchers have realized the high incidence of psychological problems of college students, the relevant research still focused on adolescents in primary and secondary schools, and social research and intervention on the depression and anxiety of college students are rare [12]. Therefore, identifying the causes of Chinese college students’ psychological issues during the epidemic is crucial for developing effective intervention strategies and enhancing their mental health. Identifying the influencing factors (such as sports activities and body image) will conduce to formulate corresponding strategies to improve the happiness of college students in China, to provide an important reference for future self-esteem education and health-promoting behaviour intervention.

### Literature review

With the development of society, under the multiple effects of internal and external factors, the level of physical activity (PA) of college students is becoming lower and lower, and their physique level is becoming depressed [13]. Under the impact of the COVID-19 epidemic, this trend has attracted more worries [14]. The positive effects of sports activities on mental health is the consensus of academia. Researches have shown that PA brings a series of physiological reactions, such as increasing the level of endorphin [15, 16], and attenuating the hypothalamic–pituitary–adrenal (HPA) axis response pressure [17]. And psychological reactions, such as depression and anxiety reduction [18, 19], improve cognitive function [20] and happiness [21] to promote mental health. However, while we are becoming more aware of the significance of PA, our knowledge of the relation between PA and depression/anxiety is not comprehensive. It has been shown that PA is negatively associated with depression and anxiety [22], while a meta-analysis concluded that PA was not the associated factors of their prevalence [23]. A new review suggests that it is controversial whether levels of PA that can reduce depression and anxiety [24]. There are still inconsistencies in the relationship between them, more evidence needs to be provided.

Given the powerful role of PA in college students’ mental health problems, people begin to explore the complex relationship between PA and depression/anxiety to effectively deal with the depression and anxiety of college students. However, the study found that the relationship between PA and depression/anxiety is not a simple direct relationship, and there are many regulatory and intermediary factors between the two, these factors may lead to inconsistency in their relationship. Previous studies have confirmed that both self-system and behavioral activation are responsible for their inconsistency [25, 26]. Although many studies have demonstrated all three of PA, body image and BMI have a strong correlation with depression/anxiety, the role played by body image and BMI in PA-depression or PA-anxiety remains a mystery. Body image and BMI may be the main cause of the inconsistencies in these relationships.

Body image refers to the multifaceted psychological experience of embodiment including one’s body-related self-perceptions and self-attitudes, such as cognition, beliefs, feelings and behaviours [27, 28]. It has been defined as a person’s “inside view” of their body-that is, their feelings, perceptions, thoughts, and beliefs about their body that impact how they behave toward it [29]. There are significant inconsistencies in the relationship between body image and PA. Some researchers believe that body image is related to physical activity levels [30]. For instance, regardless of BMI, body image was found to be a statistically positive predictor of PA for adolescents in a study on PA, BMI, and body image [31]. But others believe that there is no significant correlation between PA and body image [32, 33]. Having a poor body image might be harmful to your health [34], and this might cause psychosocial dysfunction [35], leading to low self-esteem and psychological pain [36]. Previous researches have shown that body image dissatisfaction may be associated with the production of depression and anxiety [37, 38] and has become an important public health issue [39]. However, earlier studies have focused on gender differences in body image and the effect of PA on depression and anxiety, while less considering the interference of other factors. Given the strong correlation between body image and depression/anxiety, we speculate that body image may have contributed to the inconsistency in the relationship between PA and depression/anxiety.

BMI is an objective reference for body weight which reflects the body’s fat and leanness. Numerous studies have shown that BMI and PA have a well-established relationship. Teenagers with higher physical activity levels are less likely to be obese, and have higher probability to have a normal BMI [40, 41]. Getting the right amount of PA is crucial for controlling body weight, and helps lower BMI in obese persons [42]. Previous studies focusing on female depression have shown that poor BMI is significantly associated with depression levels [43], and BMI is positively correlated with mental health and PA [44]. The relationship between BMI and body image is complicated, and poor BMI may lead to lower body image levels [45]. College students who are too emaciated or obese may be denied PA or other social interactions by their peers or escape initiatively due to a lack of athletic ability and poor appearance [46, 47], which can lead to depression and anxiety. Although many studies have recognized the effect of BMI on psychological problems such as depression and anxiety, the research has generally targeted on the direct impact of BMI on PA, depression and anxiety. And the role that BMI may have played in the relationship between PA and depression/anxiety has been ignored.

**Fig. 1:**
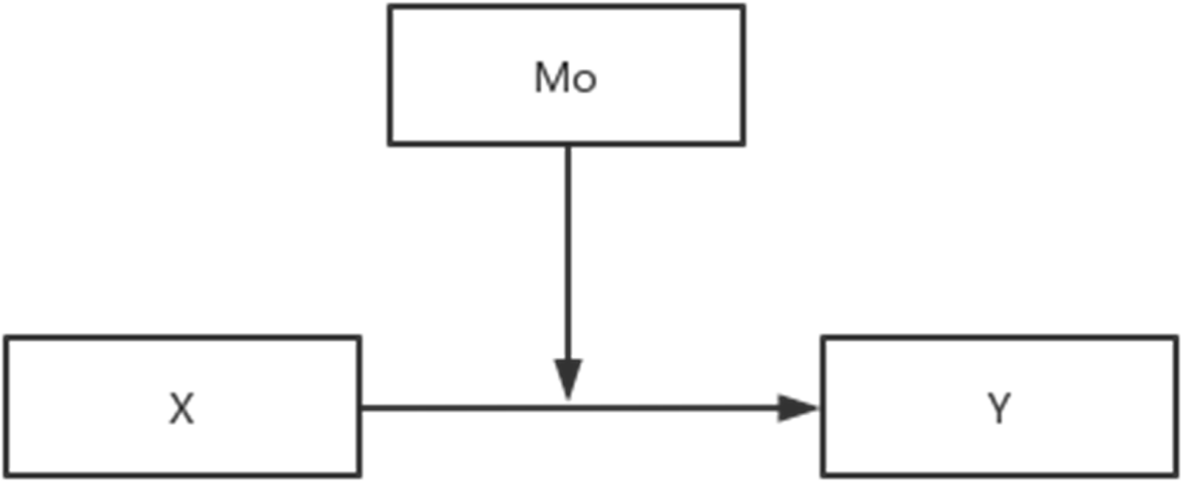
Conceptual model. Mo = body image or BMI; X = physical activity; Y = anxiety or depression

According to the Theory of Planned Behavior, sports behaviour is decided by intention, attitude, subjective cognition and evaluation [48]. Sports behaviour comes from sports motivation and intention which comes from people’s cognition, evaluation and attitude [49]. Thus, forced by the desire to improve appearance or to be thin or strong [50, 51], PA arose as a behaviour. By examining the impact of body image and BMI, the current study seeks to address inconsistencies in the relationship between PA and depression/anxiety in college students. Additionally, depression and anxiety are considered a posterior factor in this study, and it examines any potential connections between depression/anxiety and levels of PA from the perspectives of body image and BMI. The results of this study will help design self-esteem instruction and PA interventions in the future that are better able to support the physical and mental health of the adolescents in this study who are at risk. The representation of the structural model is shown as follows (Fig. 1).

## Methods

### Participants and procedures

In this study, a total of 312 college students from one college in Jinan, Shandong were selected by convenience sampling method. During the study period, the COVID-19 epidemic in China was brought under certain control, but the new cases and the negative impact of the epidemic still existed. Students at the school were under “relatively closed management” from April to May 2022, meaning that their activities were restricted to the campus. According to the research needs, an electronic questionnaire was used to conduct the survey. All researchers concerned in the study received the required formal training. Each participant completed a questionnaire containing a letter that briefly introduced the concept and motive of the study and emphasized the confidentiality and anonymity of the research. Respondents had been requested to answer all questions voluntarily and privately then submitted the questionnaires electronically once completed.

The questionnaire asks for basic sociodemographic information such as age, sex, major, hometown, grade, number of siblings, height, and weight. The trained researchers also collected data on college students’ levels of PA, body image, depression, and anxiety. The questionnaire was given to students before class. After the questionnaire was recovered, a 100% quality control check on completed questionnaires was conducted. According to the screening criteria, 61 invalid questionnaires were excluded, and 251 legitimate have been got last, with a resultful rate of 80.4%. This study was approved by the Ethics Committee of School of Public health, Shandong University (No. 20190912).

## Materials

### Physical activity

The International Physical Activity Questionnaire-Short Form (IPAQ-SF) prepared by the International Working Group on Physical Activity Measurement, was selected to investigate the physical activity levels of Chinese college students. The IPAQ has been translated into Chinese with a good reliability and validity [52]. There are 7 items in the questionnaire. In the first six questions, participants were asked about the frequency and daily cumulative time of PA in the past seven days, from the aspects of high-intensity PA, moderate intensity PA and walking. The last question asked about the sitting situation [53]. According to ‘WHO Guidelines on Physical Activity and Sedentary Behaviour’, and the amount of PA for adults recommended [54]. Those who did not meet the above standards were defined as “PA isn’t up to the standard” with at least 150 min of moderate-intensity aerobic exercise that week or at least 75 min of high-intensity physical exercise or a integration of moderate and high-intensive physical exercise per week as the demarcation.

### Body image

The Body Image Questionnaire [55] was used for the measurement of body image, which is intercepted from the Cosmetic Procedure Screening Questionnaire (COPS). The subjects’ body perception and body concept were measured from the aspects of self-body attitude, self-body perception and self-body satisfaction. The questionnaire has 12 items on a 9-point scale (e.g., “How much does your feature (s) currently cause you a lot of distress? Slabs of 1 = Not at all distressing to 9 = Extremely distressing”), the higher the score, the more negative the body image one feel. To facilitate the analysis, the raw data were inverted and transformed. The Cronbach α coefficient of the scale in this study was 0.868.

### Depression and anxiety

Self-rating Depression Scale (SDS) and self-rating Anxiety Scale (SAS) [56] were used respectively. These scales are standard assessment instruments, and their reliability and validity have been examined in Chinese population [57, 58]. Both questionnaires are 4-point Likert scale, both containing 20 items, with a score of 0–3 for each item. The scores of the depression/anxiety scale were the score of the subject in the dimension of depression/anxiety. The lower the score, the lower the level of depression/anxiety of the subject. In this study, the Cronbach α coefficients of depression and anxiety were 0.912 and 0.921, respectively.

### BMI

BMI was calculated for body mass in kilograms divided by stature in squared meters. The weight status of college students was determined according to the Chinese standard which was proposed by the Chinese Obesity Working Group [59]. The normal weight is BMI ≤ 23.9 and ≥ 18 kg/m^2^, which means they are in better shape and more likely to have a better body image. BMI ≥ 24.0 or ≤ 18.0 kg/m^2^ is overweight or thin, which means the objective body shape is poor. In the following paragraphs, BMI refers to objective body shape.

### Statistical analysis

The raw data were derived from the Wenjuanxing questionnaire platform (https://www.wjx.cn/). All the statistical analyses were performed using SPSS version 26.0. Descriptive statistics for continuous variables were expressed as means and standard deviation, and categorical variables were summarized as percentages (%). Normality of the data was checked using the Kolmogorov–Smirnov test. Spearman correlation analysis model was used to analyze the binary correlation among the variables, and the chi-square test, or variance analysis (variance analysis) was used to compare the statistical differences of college students with different demographic characteristics in each variable. Model I in Hayes’ PROCESS macro in SPSS (version 3.5) was used for the regression analysis of the moderating effect of body image (or BMI) on the relationship between PA and depression/anxiety of college students [60]. 

Finally, to test for a potential moderating effect of body image and BMI in PA-depression/anxiety problems, several separate analyses were conducted. We input sex, major (science, art), hometown, grade, and the number of siblings as the covariate, PA as an independent variable, and depression or anxiety as a dependent variable into the model. The body image-PA interaction and BMI-PA interaction were finally input to test its moderating effect [61]. The bootstrap method (5000 repeated samplings) was used to estimate a 95% confidence interval (CI) used to test the significance of the moderating effect. Before incorporating them into the moderating model, we used the original data minus its mean and then divided by standard deviation to standardize all continuous variables.

## Results

A total of 251 Chinese college students were surveyed, including 145 males (57.8%) and 106 females (42.2%). The mean (standard deviation) age of the participants was 20.66 (1.23) years. Among these samples, 129 (51.39%) participants were from rural areas, and 122 (48.61%) were from urban areas. 138 (54.98%) participants were engineering students and 113 (45.02%) majored in literature. The mean (standard deviation) of BMI (kg/m^2^), Height (m) and Weight (kg) was 21.50 (3.33), 1.73 (0.083), and 64.98 (11.37). The study found that there were only significant differences in height and weight between boys and girls.

Table 1 shows the descriptive statistics of PA, depression and anxiety of college students and their differences in control variables. The test with gender as an independent variable showed that the gender difference in physical activity levels was significant [t (251) = 4.538, *p* < 0.001], and the physical activity levels of boys was significantly higher than that of girls. In addition, the level of PA was significantly different in the distribution of disciplines [t (251) = -2.047, *p* < 0.05], and the level of PA of liberal arts students was higher. The distribution of depression among grades varied significantly [t (251) = 2.679, *p* < 0.05]. Anxiety was significantly different in the distribution of disciplines [t (251) = -2.549, *p* < 0.05]. The senior college students were more likely to depression. The anxiety levels of liberal arts students was lower.

**Table 1:** Descriptive statistics of PA, depression and anxiety of college students and their differences in covariate

Control variables (*N* = 251)	PA		Depression		Anxiety	
	M(SD)	t/F	M(SD)	t/F	M(SD)	t/F
gender						
Male *n* = 145	0.72(0.50)	4.538^***^	38.77(12.19)	-1.491	36.62(12.57)	-0.224
Female *n* = 106	0.43(0.50)		40.96(10.55)		36.95(10.12)	
hometown						
Urban *n* = 122	0.58(0.54)	-0.419	38.59(11.91)	-1.474	35.36(11.11)	-1.873
Rural *n* = 129	0.61(0.49)		40.74(11.16)		38.09(11.89)	
major						
Science *n* = 138	0.54(0.54)	-2.047^*^	38.73(10.66)	-1.460	35.07(10.07)	-2.549^*^
Art *n* = 113	0.67(0.47)		40.87(12.51)		38.83(12.93)	
Grade						
Freshman *n* = 56	0.62(0.58)	1.093	41.93(10.82)	2.679^*^	37.13(10.48)	1.785
Sophomore *n* = 58	0.55(0.50)		41.07(14.09)		38.79(14.02)	
Junior *n* = 114	0.64(0.48)		37.20(9.34)		35.15(10.03)	
Senior *n* = 22	0.45(0.51)		48.00		37.95(13.72)	
Master and above *n* = 1	0		39.69(11.56)		56.00	
Number of siblings						
None *n* = 111	0.63(0.54)	1.423	39.58(12.66)	0.012	37.79(12.78)	1.322
One *n* = 113	0.60(0.49)		39.81(11.02)		35.45(10.70)	
Tow and above *n* = 27	0.44(0.51)		39.67(9.09)		38.00(9.51)	

^*^ *p* < 0.05

^**^ *p* < 0.01

The relationship between PA, body image, depression, anxiety and BMI were showed in Table 2. PA and body image were significantly negatively correlated with depression (*r* = -0.216, *p* < 0.001), (*r* = -0.394, *p* < 0.001) and anxiety (*r* = -0.184, *p* < 0.01), (*r* = -0.403, *p* < 0.001). Depression was positively correlated with anxiety (*r *= 0.846, *p* < 0.001). PA was positively correlated with BMI (*r* = 0.302, *p* < 0.001).

**Table 2:** Correlation matrix for PA, body image, depression, anxiety, and BMI

Variable	Total (*N* = 251)	PA	BI	Depression	Anxiety	BMI
PA(%)	148 (59)	1				
BI	34.88 ± 17.04	-0.082	1			
Depression	39.69 ± 11.56	-0.216^***^	-0.394^***^	1		
Anxiety	36.76 ± 11.58	-0.184^**^	-0.403^***^	0.846^***^	1	
BMI(%)	184 (73.3)	0.302^***^	0.055	-0.081	-0.048	1

*BI* Body image. PA report the percentage of people who meet the criteria, and BMI report the percentage of normal people

^**^ *p* < 0.01

^***^ *p* < 0.001

The results in Table 3 show that in the moderating model, PA had a significantly negative effect on depression and anxiety levels (β = -0.579, *P* < 0.001; β = -0.553, *P* < 0.001), which suggest that college students who have higher level of physical activity are less likely to develop depression and anxiety. Body image significantly and negatively affected depression and anxiety levels (β = -0.492, *P* < 0.001; β = -0.574, *P* < 0.001). In addition, the interaction between PA and body image had a significant positive effect on anxiety levels (β = 0.270, *P* < 0.05). This suggest that the relation between PA and anxiety among college students is moderated by their body image. It showed that the relationship between PA and anxiety was moderated by body image.

**Table 3:** Regression results for the moderating effect of body image on PA and depression/anxiety

The regression equation		Overall fitting index		Significance of regression coefficient			
Outcome variable	prognosis variate	*ΔR*^*2*^	*F*	*β*	*95%CL*		*t*
Depression	PA	0.234	10.680^***^	-0.579	-0.803	-0.355	-5.089^***^
	BI			-0.492	-0.664	-0.321	-5.650^***^
	PA*BI			0.170	-0.050	0.389	1.519
Anxiety	PA	0.265	12.333^***^	-0.553	-0.773	-0.334	-4.962^***^
	BI			-0.574	-0.742	-0.406	6.724^***^
	PA*BI			0.270	0.054	0.485	2.466^*^

All continuous variables in the model are standardized and then put into the regression model; BI: body image; CI: confidence interval

^*^ *p* < 0.05

^**^ *p* < 0.01

^***^ *p* < 0.001

Table 4 showed that BMI had no moderating effect on the relationship between PA and depression (anxiety) (*p* > 0.05).

**Table 4:** Regression results for the moderating effect of BMI on PA and depression/anxiety

The regression equation		Overall fitting index		Significance of regression coefficient			
Outcome variable	prognosis variate	*ΔR*^*2*^	*F*	*β*	*95%CL*		*p-Value*
Depression	PA	0.074	3.641^***^	-0.505	-1.001	-0.009	0.046
	BI			-0.122	-0.498	0.254	0.523
	PA*BI			0.009	-0.558	0.577	0.974
Anxiety	PA	0.079	3.732^***^	-0.324	-0.819	0.172	0.200
	BI			-0.046	-0.422	0.330	0.809
	PA*BI			-0.183	-0.750	0.384	0.525

*CI* Confidence interval

^***^ *p* < 0.001

**Fig. 2:**
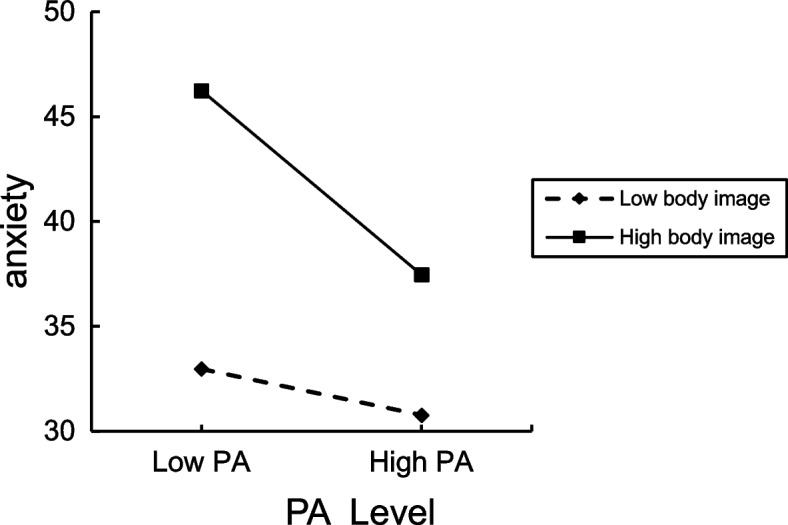
Moderating effect of body image on the PA-anxiety relationship

We tried to further elucidate the moderating effect of body image between PA and anxiety. In this study, we defined the grouping of body image as follows. High was the group with a body image score higher than the average plus one standard deviation; low was the group with a body image score lower than the average minus one standard deviation. The simple slope test was carried out by the point selection method [62], and the interaction effect was shown in Fig. 2. The contribution made by moderating effect to the variance was 1.61% (ΔR^2^ = 0.0161, *p* < 0.05). Simple slope test showed that the relationship between PA and anxiety decreased with the increase in body image levels. That is, PA is more strongly correlated with anxiety in people with high body image.

## Discussion

College students’ mental health has been widely concerned by the community, especially depression and anxiety—the most serious and common problems among college students during the COVID-19 Pandemic [63]. The current investigation explored the relationship between PA, BMI, body image, depression and anxiety of college students, and verified whether body image and BMI play a regulatory role in the relationship between PA and depression/anxiety of Chinese college students. PA was negatively correlated with depression and anxiety, and positively correlated with BMI. Body image was negatively correlated with depression and anxiety, but not significantly with PA. Body image moderated the relationship between anxiety and PA, but BMI did not. Gender differences in physical activity levels are significant, with boys having higher levels of PA than girls. In addition, liberal arts students have higher physical activity levels and lower anxiety. Which may be due to their relatively small academic pressure and relatively abundant time. There are significant differences in the distribution of depression in grades. Higher grade students have higher levels of depression, which may be caused by more academic stress and work anxiety [64].

This study shows that college students’ PA is positively correlated with depression and anxiety. College students who have reached the standard of PA show lower levels of depression and anxiety. The positive effect of PA on psychological problems such as depression and anxiety is an undoubted consensus in academia [65]. We conjecture that college students with high PA levels not only get physiological benefits from PA but also participate more often in social intercourses [66, 67]. Through PA they improved their ability to resist setbacks and became more extroverted, thereby reducing the degree of depression and anxiety. At the same time, college students with depression and anxiety often show introversion and low sociality [68, 69], which also reversely leads to the reduction of PA.

In addition, we found that the body image of college students had a moderating effect on PA and anxiety, but not on depression. Dissatisfaction with one’s body image is a common problem among college students, especially female college students [70]. It has also been found to be highly correlated with social body anxiety [71]. The results show that with the increase in body image levels, the relationship between PA and anxiety strengthens. This may be because body image itself and anxiety are significantly related [72], as important factors. Body image plays a synergistic role in reducing anxiety with PA, so the more positive the body image is, the better the effect of PA on reducing anxiety is. Furthermore, our study find that college students’ body image is significantly correlated with depression and anxiety, which may be due to mentally healthy college students having a better knowledge of their bodies. Unfortunately, our results show that although the correlation between body image and anxiety is higher than that of depression, the difference is small. The association between PA, body image, depression and anxiety should be further explored through longitudinal or interventional studies in the future.

The results show that there is no significant correlation between college students’ body image and PA [32, 33]. However, some scholars hold other views. Korn believe that PA has a significant predictive function on a body image levels [30]. Zaker and Radzi’s study demonstrates that PA is weakly correlated with body image in female groups [73]. The reasons for the controversy may be the different groups and methods of the survey, such as gender has significant differences in PA and body image. Males have higher physical activity levels, and females show more dissatisfaction and anxiety with their bodies [74], and the influence of other variables cannot be excluded by single factor analysis. In addition, our subjects were Chinese college students who were closed during the epidemic prevention and control. Under the background of the epidemic, the uncertainty factors increased, and the PA and physical activity space of college students were limited, which affected the physical activity levels of college students. In the future, scholars should observe whether there is a correlation and causality between PA and the body image of college students through longitudinal experiments.

At the same time, our study only found that BMI (normal/abnormal, the state of abnormal includes the obesity and weight loss) was positively correlated with PA, which proves that PA is a key factor affecting the body shape of college students, and college students with higher physical activity levels are more likely to have good external shape. However, there is no moderating effect of BMI on the results. Therefore, combined with the moderating role of body image on the relationship between PA and anxiety, it is reasonable to believe that subjective perception and evaluation play a decisive role in the relationship between PA and anxiety compared to the objective body image. Additionally, it is worth noting that BMI does not correlate with depression and anxiety, while body image has a significantly negative correlation with them. The study suggests that some college students may be depressed and anxious due to the lack of correct understanding of the body and the existence of physical anxiety and other negative emotions. However, the negative body shape does not necessarily lead to the negation and wrong understanding of the self-body [37]. Therefore, it is necessary to guide college students to correctly understand their bodies and maintain a positive attitude toward them.

Some limitations of this study should be noted. Firstly, the physical activity levels of college students during the COVID-19 pandemic prevention and control may be different from that in normal times [74, 75], and the depression and anxiety are more acute [4]. Secondly, although this research has carried on the sample screening, the university student self-report data may still exist deviation; in addition, causal reasoning is impossible taking into account the cross-sectional nature of this study (and there are likely to be some reverse causal relationships), which cannot further analyze the lead-lag relationship between variables. However, these findings emphasize the necessity to take into account body image in PA interventions and provide early evidence. Third, the sample size of this study is small, and the study population is convenient samples from universities rather than clinical samples. Our conclusions from these healthy or non-painful populations may be only applicable to healthy or less depressed/anxious students, and not necessarily applicable to clinical or pain-based samples. Fourthly, the analyses were conducted on a mixed-gender sample, while the concerns of body image may be gender-specific. Finally, the study suggests that in addition to body image and BMI and the motivation of previous studies, other factors affecting the relationship between PA and depression/anxiety still need to be explored or confirmed. Future research is needed to conduct longitudinal experiments to explore the specific mechanism of PA and depression/anxiety to find other variables with regulatory or intermediary effects.

Despite these limitations, this study has several strengths. To our knowledge, this is the first study to discuss the moderating effect of body image and BMI on the associations between PA and depression/anxiety. We learn the relationship between PA and depression/anxiety from a new perspective and explain the inconsistencies of the relationship in previous research from both subjective and objective perspectives. The body image is used to reflect the subjective evaluation of college students’ body shape, and BMI is used to reflect the fatness and thinness of the body. The conclusion is, subjective cognition and attitude seem to be of more momentous significance than objective image when discussing the relationship between PA and depression/anxiety. The study enriches the research on the relationship between PA, depression and anxiety and provides a theoretical basis for the necessity of self-esteem education. In addition, the results of this study also proved the necessity of establishing a correct understanding of college students’ self-body image, reminding the relevant personnel to pay attention to the establishment of relevant courses. Furthermore, we can also try to carry out body image education in the process of sports intervention for college students with depression or anxiety, which may obtain better results.

## Conclusion

As we know, this is the first study to examine the relationship between PA, depression, anxiety, body image and BMI of Chinese college students during the COVID-19 epidemic. The findings provide preliminary evidence that PA has a positive effect on depression and anxiety. Therefore, it is necessary to encourage college students to participate in various types of physical activities. Body image has a high correlation with depression and anxiety and has a significant indigenous moderating effect on the relationship between PA and anxiety. At the same time, the study has proved that the common hypothetical object (BMI) of the influencing factors of depression/anxiety has no significant indigenous correlation with depression and anxiety, and it has no moderating effect on the relationship between PA and depression/anxiety. Therefore, we make the following inference: compared with external forms, it is more important to change the cognitive level and attitude of college students toward their bodies. In the future, when we are exercising intervention for depression and anxiety college students, attention should be paid to self-esteem education related to physical cognition.

## Data Availability

The datasets used and/or analyzed during the current study are available from the first author upon reasonable request.

## Abbreviations

PA: Physical activity
BMI: Body mass index
